# Supplementary material for: Learning curve and surgical outcome of robotic assisted colorectal surgery with ERAS program
Source: Sci Rep. 2022 Nov 29;12:20566. doi: 10.1038/s41598-022-24665-w (PMC9709162; doi:10.1038/s41598-022-24665-w)
Supplement: Supplementary file 2 — Supplementary Information 2. [file 41598_2022_24665_MOESM2_ESM.zip › RAL Raw data-Ñ[▒K/054/Patho_ñw╝╨░O▒Kñσ.pdf]

臺中榮民總醫院　臺中市臺灣大道四段1650號　病理部活體組織切片　臨床醫學實驗及病理診斷中心 檢驗(查)報告

[redacted]

Pathologic diagnosis:
1. Cyst, liver, unroofing -- Biliary cystadenoma.
2. Sigmoid colon, robotic anterior resection --- Diverticulosis.
3. IMA root, dissection --- Unremarkable fibrovasculoadipose tissue.
4. Colon, distal cut end, resection --- Congestion.

Ancillary study for diagnosis:
Immunohistochemical stains for CK7 and CK19 done in section A. The cystic lining cells are immunoreactive to both CK7 and CK19.

Gross description:
The specimen consists of 1) 2 pieces of ruptured cystic tumor, up to 7x2.8x1 cm, labeled as "liver cyst". The wall of cyst is thin and without obvious inner content. 2) a segment of colon measures 11 cm in length, 2.5 cm in circumferential of proximal cut end, and 4 cm in circumferential of distal cut end. There are multiple small diverticulum noted at the mucosa. Other parts of specimen are unremarkable. 3) a piece of tan soft tissue, 1.8x1x0.5 cm, labeled as "IMA root". 4) a piece of tan soft mucosa-covered tissue, 2.3x2x1.3 cm, labeled as "distal cut end". Representative parts for section: A-B) liver cyst C-D) bilateral cut ends E-J) lesion; All for section: K-L) specimen 3-4.
#T-59470_2 #M-32700_2 0 1100 000000
#T-62000_2 #M-81610_2 0 1100 000000

已電子簽章

敬告：本單僅供醫師參考使用，不具任何申請效力！！
請病患妥為保管，若不慎遺失需自負個人資料外洩責任！
